# Diagnostic accuracy of methacholine challenge tests assessing airway hyperreactivity in asthmatic patients - a multifunctional approach

**DOI:** 10.1186/s12931-016-0470-0

**Published:** 2016-11-17

**Authors:** Richard Kraemer, Hans-Jürgen Smith, Thomas Sigrist, Gabi Giger, Roland Keller, Martin Frey

**Affiliations:** 1 Department of Clinical Research, University of Berne, Kirchenfeldstrasse 74, CH-3005 Berne, Switzerland; 2 Medical Development, Research in Respiratory Diagnostics, Berlin, Germany; 3 Division of Pulmonary Medicine, Clinic Barmelweid, CH-5017 Barmelweid, Switzerland

**Keywords:** (1) Airway hyperreactivity, (2) Bronchial asthma, (3) Methacholine challenge test, (4) Whole-body plethysmography, (5) Effective, specific airway conductance, (6) Diagnostic accuracy, (7) Dysanapsis

## Abstract

**Background:**

There are few studies comparing diagnostic accuracy of different lung function parameters evaluating dose–response characteristics of methacholine (MCH) challenge tests (MCT) as quantitative outcome of airway hyperreactivity (AHR) in asthmatic patients. The aim of this retrospectively analysis of our database (Clinic Barmelweid, Switzerland) was, to assess diagnostic accuracy of several lung function parameters quantitating AHR by dose–response characteristics.

**Methods:**

Changes in effective specific airway conductance (sG_eff_) as estimate of the degree of bronchial obstruction were compared with concomitantly measured forced expiratory volume in 1 s (FEV_1_) and forced expiratory flows at 50% forced vital capacity (FEF_50_). According to the GINA Guidelines the patients (*n* = 484) were classified into asthmatic patients (*n* = 337) and non-asthmatic subjects (*n* = 147). Whole-body plethysmography (CareFusion, Würzburg, Germany) was performed using ATS-ERS criteria, and for the MCTs a standardised computer controlled protocol with 3 consecutive cumulative provocation doses (PD_1_: 0.2 mg; PD_2_: 1.0 mg; PD_3_: 2.2 mg) was used. Break off criterion for the MCTs were when a decrease in FEV_1_ of 20% was reached or respiratory symptoms occurred.

**Results:**

In the assessment of AHR, whole-body plethysmography offers in addition to spirometry indices of airways conductance and thoracic lung volumes, which are incorporated in the parameter sG_eff_, derived from spontaneous tidal breathing. The cumulative percent dose-responses at each provocation step were at the 1^st^ level step (0.2 mg MCH) 3.7 times, at the 2^nd^ level step (1 mg MCH) 2.4 times, and at the 3^rd^ level step (2.2 mg MCH) 2.0 times more pronounced for sG_eff_, compared to FEV_1_. A much better diagnostic odds ratio of sG_eff_ (7.855) over FEV_1_ (6.893) and FEF_50_ (4.001) could be found. Moreover, the so-called dysanapsis, and changes of end-expiratory lung volume were found to be important determinants of AHR.

**Conclusions:**

Applying plethysmographic tidal breathing analysis in addition to spirometry in MCTs provides relevant advantages. The absence of deep and maximal inhalations and forced expiratory manoeuvres improve the subject’s cooperation and coordination, and provide sensitive and differentiated test results, improving diagnostic accuracy. Moreover, by the combined assessment, pulmonary hyperinflation and dysanapsis can be respected in the differentiation between “asthmatics” and “non-asthmatics”.

**Electronic supplementary material:**

The online version of this article (doi:10.1186/s12931-016-0470-0) contains supplementary material, which is available to authorized users.

## Background

Airway hyperreactivity (AHR) is a characteristic feature of bronchial asthma, and methacholine challenge testing (MCT) is well established to quantitate AHR in patients with unexplained symptoms such as cough, chest tightness and/or dyspnea, when the diagnosis of asthma is uncertain [1–10]. Compared to control subjects the provoked bronchial obstruction appears earlier and at lower provocation doses, and is more intensive in patients with asthma, a functional feature serving as rationale for the underlying mechanisms of AHR [4, 11, 12]. Both the European Respiratory Society (ERS) [1] and the American Thoracic Society (ATS) [4] recommend bronchial provocation tests by inhalation of aerosolized methacholine (MCH), considering this approach to be a reproducible and relatively easy to perform test in adults and children.

Following MCT, the cumulative provocation dose (PD) at which the forced expiratory volume in 1 s (FEV_1_), measured with spirometry, decreases at least 20% compared to baseline (PD_−20_FEV_1_), is currently the most commonly used outcome of assessing AHR by detection of flow limitation in consequence of airway narrowing [9]. Alternatively, whole-body plethysmography can also be used to determine AHR, measuring changes in airway mechanics either by a 35% [2, 7], a 40% [4, 5], or a 50% [5] reduction in specific airway conductance (sG_aw_) or an increase of specific airway resistance (sR_aw_) of 100% [8, 10] from baseline. To our knowledge, studies comparing reliability of spirometric parameters with those obtained by whole-body plethysmography, or even a combination of both are rare, and the so-called effective specific airway conductance (sG_eff_) has never been evaluated as a target parameter.

Therefore, the purpose of the present study was to compare diagnostic value of the response characteristics to MCH assessed by FEV_1_ and forced expiratory flow at 50% of vital capacity (FEF_50_), in relation to sG_eff_ differentiating different diagnostic and functional groups and to encounter possible differences of AHR in relation to pulmonary hyperinflation and/or the phenomenon of dysanapsis [13–17].

## Methods

### Study population and ethics

The study was conducted in the Division of Pulmonary Medicine, Clinic Barmelweid, CH-5017 Barmelweid, Switzerland, recruiting patients referred to the pulmonary function laboratory for baseline pulmonary function and MCT in patients with symptoms suggestive for asthma, such as cough, shortness of breath, wheezing or chest tightness. According to the classical definition of the GINA dissemination committee report [18], and based on the characteristics of the case histories and clinical findings, two experienced pulmonary physicians (Co-authors MF and TS) have classified the cases previously into a group of patients with proven bronchial asthma (*n* = 337; 69.8%), and a group of non-asthmatic subjects (*n* = 147; 30.4%). *Bronchial asthma* was diagnosed when subjects presented with a medical history of atopy (allergic rhinitis, hay fever, exercise-induced, partly also an eosinophilic inflammation, infection-induced, occupational or intrinsic), and documented with a positive MCT (PD_−20_FEV_1_). The groups of *non-asthmatic subjects* was composed of 4 subgroups according to the potential origin of pulmonary symptoms differentiating either between (*i*) an “upper airway syndrome” (UACS), previously also termed as post-nasal-drip-syndrome [19] (*n* = 55; 37.4%), (*ii*) a gastroesophageal reflux disease (GERD, *n* = 40; 27.2%), (*iii*) “persistent chronic cough” lasting more than 8 weeks [20] (*n* = 30; 20.4%), and patients with (*iv*) a “symptom complex” such as unexplained dyspnoea, chest tightness, hyperventilation, or somatisation (*n* = 22; 15.0%), which could not be attributed to a clear diagnosis. There were no patients with chronic obstructive pulmonary disease (COPD), bronchiectasis, cystic fibrosis, obstructive sleep apnoea syndrome (OSAS), or interstitial lung disease. Short-acting ß_2_-agonists were withdrawn for 8 h, long-acting beta-agonists for 48 h, and leukotriene receptor antagonists for 24 h prior to the lung function testing. Inhaled corticosteroids were withdrawn 7 days before MCT.

We conducted this study retrospectively as a case controlled study in order to compare diagnostic accuracy of the MCT assessed by different target parameters between the diagnosed asthmatic patients and non-asthmatic subjects. Inclusion criteria were reproducible base-line measurements, at least 5 plethysmographic tidal breath efforts as well as at least 3 reproducible forced expiratory manoeuvres at each provocation level. The study was planned according to the Federal Law of Human Research, conceptualized according to the Swiss Ethics Committee on Research involving humans, and approved by the Governmental Ethics Committee of the State of Berne. Master-files haven been stored and secured in the REDCap-system of the Clinical Trial Unit, Medical Faculty, University of Berne, Switzerland.

### Pulmonary function procedures

Spirometry and plethysmographic measurements were performed using standard techniques according to ATS-ERS recommendations [21, 22] previously established and subsequently extended (http://www.atsjournals.org/doi/suppl/10.1164/rccm.200407-948OC/suppl_file/online_methods.pdf) [23]; http://www.atsjournals.org/doi/suppl/10.1164/rccm.200603-423OC/suppl_file/onlinesup200603-423ocr2.pdf) [24]; http://www.biomedcentral.com/content/supplementary/1465- 9921-10-106-S1.doc) [25].

Using a Jaeger MasterLab whole-body plethysmograph (CareFusion, Würzburg, Germany), measurements of airway mechanics, slow spirometry and hence assessment of static lung volumes were carried out first and only afterwards forced flow-volume loops were performed according to the ATS-ERS recommendations (best value of the 3 trials). The sR_eff_, and its reciprocal value the sG_eff_ resp., were assessed while the subject breathed tidally, in an upright position, through filter and measuring head in the body plethysmograph [26], without requiring any special breathing manoeuvres or efforts against a closed shutter. Both measurement techniques (whole-body plethysmography and spirometry) were performed with the subjects in sitting position within the whole-body plethysmograph cabin.

### Assessment of airway mechanics

**Fig. 1:**
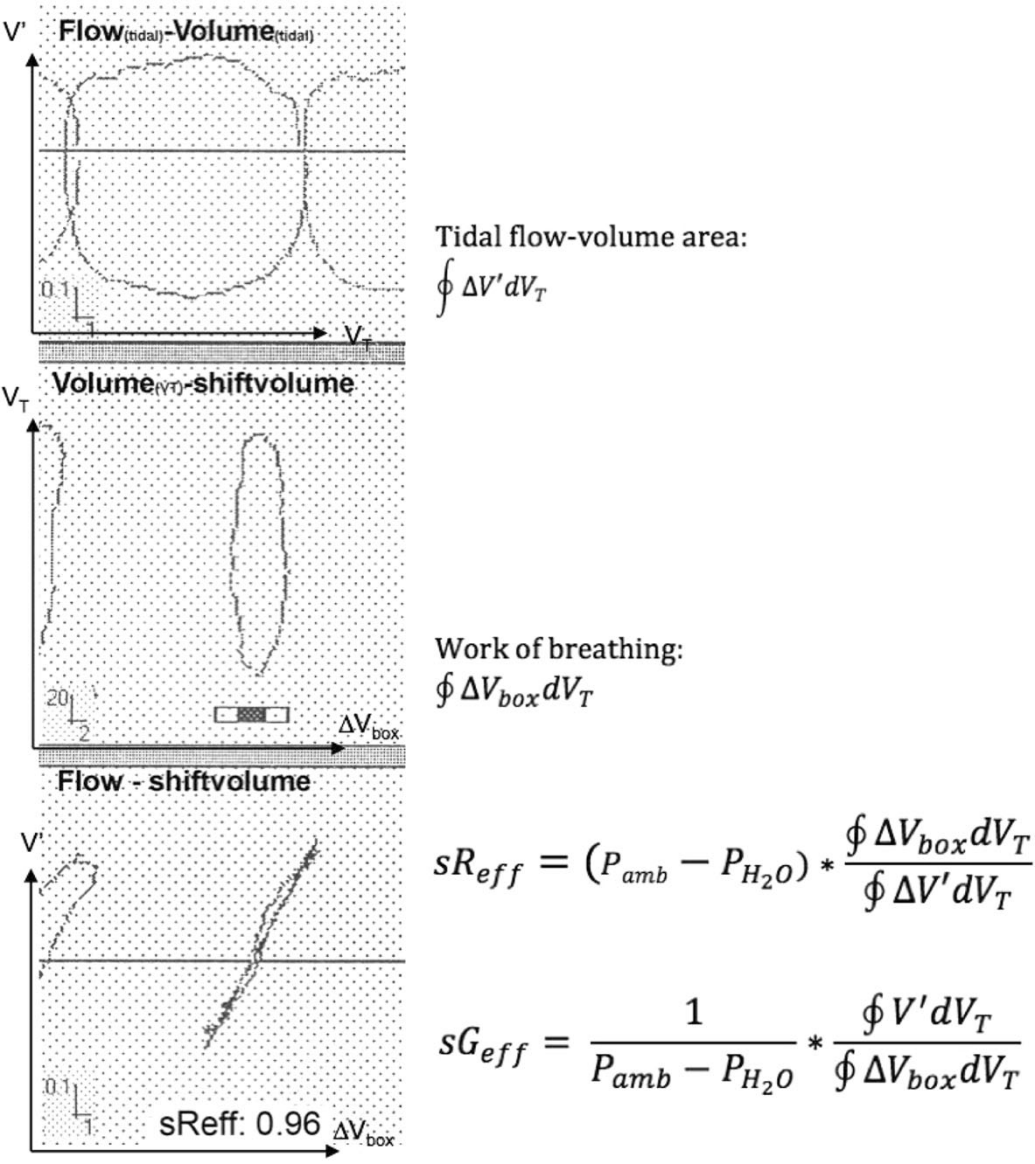
Print-screen, originally depicted from the Jaeger infant whole-body plethysmograph showing breath-by-breath tracings from which effective specific resistance (sR_eff_), and its reciprocal value, the effective, specific airway conductance (sG_eff_) are computed using the integral of the tidal flow-volume area (upper tracing) and the integral of the plethysmographic shift-volume versus tidal volume area, the latter representing the specific resistive work of breathing (middle tracing). The crossbar clearly demonstrates that the zero-flow points are perfectly in phase

In order to obtain a parallel synoptical presentation of the MCT, we routinely present the reaction of FEV_1_ and FEF_50_ together with sG_eff_. sG_eff_ is computed as the ratio between the integral of the area of the *tidal flow-volume loop* as numerator (∮*V*′*dV*_*T*_) and the integral of the area enclosed by the specific resistive work of breathing (sWOB = ∮Δ*V*_*box*_ *dV*_*T*_) [27] according the equation:

$$ s{G}_{eff} = \frac{1}{P_{amb}-{P}_{H_2O}}\ast \frac{{\displaystyle \oint }{V}^{\prime }d{V}_T}{{\displaystyle \oint}\Delta {V}_{box}d{V}_T} = \frac{1}{s{R}_{eff}} $$

where P_amb_ is the barometric pressure, P_H2O_ the saturated vapour water pressure at body temperature. The mathematical background to obtain sG_eff_ as the reciprocal value of the effective specific resistance (sR_eff_), has been given previously [23–25, 28], and is presented synoptically in Fig. 1. An Additional file 1 furthermore provides details regarding the paradigm shift in the assessment of airway mechanics. During quiet breathing at end-expiratory lung volume (EELV) measurements of sG_eff_ were measured in a first phase of testing. In a second phase of testing functional residual capacity (FRC_pleth_) was assessed by normal resting breathing against the closed shutter (no panting). In a third phase a slow maximal vital capacity manoeuvre, carefully linked with the shutter-measurement was performed to get the static lung volumes, functional residual capacity (FRC_pleth_), residual volume (RV) and total lung capacity (TLC). However, only in a forth phase of testing 3 consecutive forced flow-volume-loops were recorded. The median of at least 5 consecutive single measurements of sG_eff_ and 3 measurements of FRC_pleth_ were computed_,_ and from the triplet of forced expiratory manoeuvres the best effort was analysed. The pulmonary function test data were expressed as a percentage of predicted normal values, and z-transformed accordingly [21, 29, 30].

The assessment of the dose–response characteristics to MCH, observed not only by forced spirometry, but additionally by whole-body plethysmography, was chosen because a certain paradigm shift at least in the technical approach of the assessment of airway mechanics is established since several years, albeit the combined assessment of AHR by MCH by spirometry and whole-body plethysmography is not yet well validated and established in its clinical use. However, improved reliability of test results can be expected by assessing the bronchial reaction to sG_eff_, which is computed real-time by an integral, multi-dimensional approach. Details are given in the Additional file 1. Our anticipated advantages, therefore, were to elaborate, whether or not in comparison to the spirometric approach, the new plethysmographic technology could provide (*i*) better diagnostic accuracy, (*ii*) leading by independency of deep inspirations and hence changes in the volume-history [10, 31–36] to less “a priori” modulation of the airway calibre, moreover, (*iii*) by independency from the subject’s cooperation (need of forced breathing manoeuvres) circumvention of inadvertent change of the airway responsiveness during test procedure [34, 37, 38].

### The aerosol provocation system (APS)

MCT was performed according to a protocol routinely used in several pulmonary function laboratories in Europe, applying a modified dose oriented, single concentration sequence [39] with the Aerosol Provocation System (APS) using the SideStream nebulizer of Philips/Respironics previously described [38, 40]. Technical details and advantages of the APS are given in the Additional file 2, presenting the similar physical characteristics likewise they were recently outlined by Kannan et al. [41]. In their novel efforts they defined high fidelity computational simulations, performed over several breathing cycles, to get information regarding regional deposition for different particle sizes and an algorithm accounting for the re-entry of particles during the exhalation phase.

### Methacholine challenge test (MCT)

After baseline measurements, MCH (5%) was administered in 3 steps of increasing dose. However, in contrast to a previously proposed 4-step procedure [39], a one-concentration-3-step protocol with increasing single doses of MCH (0.2 mg, 0.8 mg, 1.2 mg) was performed, consisting of 3 consecutive levels of cumulative doses defined as CD_1_: 0.2 mg; CD_2_: 1.0 mg; CD_3_: 2.2 mg. The measurements of whole-body plethysmography and spirometry were performed two minutes after each MCH-inhalation. On the basis of the inhalation time and the nebulizer output provided by the APS and the known concentration of the MCH, the applied doses of MCH was individually computed at each provocation level. Response to MCH was assessed by linear regression against the logarithmic doses of MCH giving the individual PD causing either a 20% decrease of FEV_1_ (PD_−20_FEV_1_), a 40%, 45% or 50% decrease of sG_eff_ (PD_−40_sG_eff_, PD_−45_sG_eff_, PD_−50_sG_eff_, resp.), or a 20% decrease of FEF_50_ (PD_−20_FEF_50_). A synopsis of the assessment of AHR by this technique is presented in the Additional file 3. Break off criterion were when a decrease in FEV_1_ > 20% was reached or symptoms occurred. After having defined PD_−40_sG_eff_as the most discriminating factor and in order to distinguish the degree of AHR to MCH, patients were classified into 4 functional severity groups: (1) non-reactive if PD_−40_sG_eff_ ≥ 2.0 mg, (2) low-reactive if PD_−40_sG_eff_ ≥ 1.0 mg but < 2.0 mg, (3) medium-reactive if PD_−40_sG_eff_ ≥ 0.2 mg but < 1.0 mg, or (4) severe AHR, if PD_−40_sG_eff_ < 0.2 mg. Each provocation test was terminated with 2 puffs of salbutamol inhaled from spacer device.

### Data analysis and statistical methods

The discriminative power of each lung function parameter was evaluated by measures of diagnostic accuracy such as sensitivity, specificity, positive predictive values (PPV), negative predicting values (NPV), likelihood ratios (LR+; LR-), the area under receiver operating curves (ROC), the Youden’s index (J) and diagnostic odds ratios (DOR). Statistical comparisons were performed applying McNemar testing. Using these different statistical procedures several aspects of diagnostic accuracy, such as predictive ability and/or discriminative property of the MCT, could be evaluated [42]. The Additional file 4 (Statistical Approach) provides details of all the statistic methods applied. Diagnostic accuracy was tested in a first step by cross-tabulation comparing the proportions of positive and negative reaction to the MCH challenge for each parameter (sG_eff_, FEV_1_ and FEF_50_) using Chi-squared tests. Statistical analysis was performed with the IBM SPSS version 24.0 (SPSS Inc., Chicago, IL). The limit of significance was a *p*-value of 0.05.

## Results

### Patient characteristics

A total of 484 patients (199 males, 51.1%; 285 females, 58.9%; age-range 9.11–87.1 years) were eligible for inclusion in the study stratifying patients into a group of asthmatic patients (*n* = 337; 69.6%) and non-asthmatic subjects (*n* = 147; 30.4%) previously diagnosed. Table 1 shows that in the gender distribution, more females than males were found, especially in the group of asthmatics. Non-asthmatic subjects were slightly older than asthmatics. The distribution within age-classes (not shown) revealed that the younger the collective, the more asthmatics, and the older the collective, the more non-asthmatic subjects were found. The stratification into different functional groups assessed at baseline prior to MCT shows for plethysmographic measurements normal lung function in 83.1% of asthmatic patients and 80.3% of non-asthmatic subjects. Interestingly, a considerable percentage of patients presented with a pulmonary hyperinflation (FRC_pleth_ > +1.645 SD), without or in combination with bronchial obstruction (sG_eff_ < −1.645 SD). Regarding spirometry, normal flow-volume curve indices were found in 90.5% of asthmatic patients and 93.9% of non-asthmatic subjects. Only a very minor percentage of asthmatic patients (7.7%) presented with flow limitation (FEV_1_ < −1.645 SD) at baseline.

**Table 1:** Subject’s characteristics (*N* = 484; 199 males (41.1%, 285 females (58.9%), age-range (9.1–87.1 years

	Asthmatic patients *n* = 337	Non-asthmatic subjects *n* = 147				
		Upper airway Cough syndrome (UACS)	Gastrooesophageal reflux disease (GERD)	Chronic Cough (smoking, post-infection, unknown)	Symptom complex (dyspnea, chest tightness, hyperventilation, somatisation)	All non-asthmatic subjects
n (% distribution)	337 (69.6)	55 (37.4)	40 (27.2)	30 (20.4)	22 (15.0)	147 (30.4)
Gender male/female (% distribution)	129/208 (38.3/61.7)	26/29 (47.3/52.7)	18/22 (45.0/55.0)	18/12 (60.0/40.0)	8/14 (36.4/63.6)	70/77 (47.6/52.4)
Age in years (mean ± SD) (age-range)	41.3 ± 18.9 (9.1-87.1)	47.0 ± 17.0 (10.6-80.5)	55.0 ± 15.3 (26.3-83.1)	55.2 ± 17.3 (15.9-82.8)	40.4 ± 18.4 (13.9-72.9)	49.9 ± 17.5 (10.6-82.8)
Functional Characteristics of plethysmographic measurements assessed by z-scores n (% within group)						
Normal LF	280 (83.1)	42 (76.4)	31 (77.5)	26 (86.7)	19 (86.4)	118 (80.3)
Pulmonary hyperinflation (PHI)	28 (8.3)	12 (21.8)	7 (17.5)	2 (6.7)	3 (13.6)	24 (16.3)
Bronchial obstruction (O)	26 (7.7)	1 (1.8)	2 (5.0)	0	0	3 (2.0)
PHI and O	3 (0.9)	0	0	2 (6.7)	0	2 (1.4)
Functional Characteristics of spirometric measurements assessed by z-scores by plethysmography n (%)						
Normal F-V curve	305 (90.5)	50 (36.9)	40 (100)	29 (96.7)	19 (86.4)	138 (93.9)
Flow limitation (FL)	26 (7.7)	5 (9.1)	0	1 (3.3)	3 (13.6)	9 (6.1)
SAD	1 (0.3)	0	0	0	0	0 (0)
FL & SAD	5 (1.53)	0	0	0	0	0

*LF* lung function, *PHI* pulmonary hyperinflation (FRCpleth > 1.645 SD), *O* airway obstruction (sGeff < 1.645 SD), *F-V* flow-volume, *FL* flow-limitation (FEV1 < −1.645 SD), *SAD* small airway dysfunction (FEF50 < − 1.645 SD)

### Dose–response curves obtained by using different lung function parameters

**Fig. 2:**
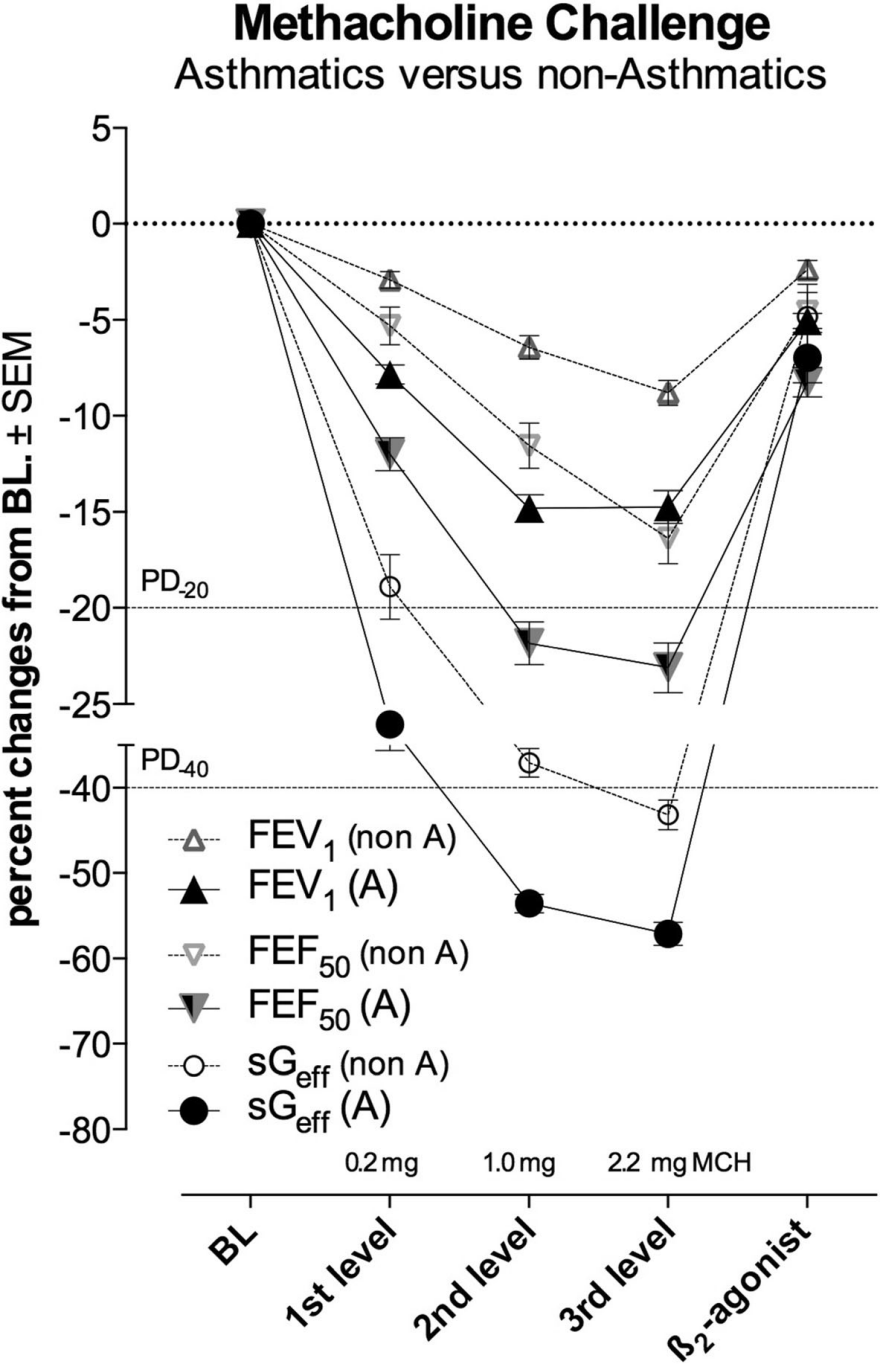
Dose–response curves of methacholine at each consecutive provocation levels (PD_1_: 0.2 mg; PD_2_: 1.0 mg; PD_3_: 2.2 mg methacholine and reversibility to a ß_2_-agonist) referred for effective specific conductance (sG_eff_), forced expiratory volume in one second (FEV_1_) and forced expiratory flow at 50% vital capacity (FEF_50_) in relation to the PD-level −20% (PD_-20_) for FEV_1_ and FEF_50_, PD-level −40% (PD_-40_) for sGeff resp., regarding asthmatic patients (A = in comparison with non-asthmatic subjects (non-A)

In Fig. 2 the dose-responses to MCH at each provocation level, expressed as percent changes from baseline (BL set to zero) for each lung function parameter are synoptically presented, comparing response in asthmatic patients with non-asthmatic subjects. It can be shown that the response assessed by sG_eff_ was much quicker and much more pronounced than the response assessed by FEV_1_ or FEF_50_. The cumulative percent-responses at each cumulative dose- (CD)-level demonstrate completely different response characteristics regarding the 3 target parameters. In asthmatic patients the cumulative percent-response of sG_eff_ was at the 1^st^ PD-level (0.2 mg MCH) 3.7 times more pronounced than with FEV_1_, 1.6 times than with FEF_50_ resp. At the 2^nd^ CD-level (1 mg MCH) the percent-response of sG_eff_ was 2.4 times more pronounced than with FEV_1_, 1.4 times more than with FEF_50_ resp., and at the 3^rd^ CD-level (2.2 mg MCH) the percent-response of sG_eff_ was 2.0 times more pronounced than with FEV_1_, 1.3 times more than with FEF_50_ resp. By nature the percent-response in non-asthmatic subjects was 1.5 to 2.9 times lower than in asthmatic patients, but the differences in percent-responses between the lung function parameters were even more pronounced.

### Diagnostic accuracy of assessing AHR by using different lung function parameters

All indices contributing to the assessment of diagnostic accuracy (Additional file 4: Statistical Approach) are summarized in Table 2, differentiating between asthmatic patients and non-asthmatic subjects. Since the cut-off level for sG_eff_ is not yet clearly defined, we first compared the 3 potentially valuable thresholds PD_−40_sG_eff_, PD_−45_sG_eff_, and PD_−50_sG_eff_. Optimal determination as represented by the highest sensitivity, highest negative predictive values, highest diagnostic odds ratios (DOR) [43], lowest negative likelihood ratios, and highest diagnostic effectiveness was found for PD_−40_sG_eff_, indicating that PD_−40_sG_eff_ is the most appropriate threshold for evaluating AHR by sG_eff_. Whereas the PD_−40_sG_eff_ level was reached in all subjects by sG_eff_, the PD_−20_FEV_1_ was not reached by FEV_1_ in 9.1% of subjects and in 6.4% PD_−20_FEF_50_ by FEF_50_ respectively. Therefore, PD_−40_sG_eff_ measures AHR in comparison with PD_−20_FEV_1_ and PD_−20_FEF_50_ with better properties of diagnostic accuracy.

**Table 2:** Measures of MCH-challenge procedures computed for response characteristics of sG_eff_, FEV_1_ and FEF_50_

	PD_-40_sG_eff_	PD_-45_sG_eff_	PD_-50_sG_eff_	PD_-20_FEV_1_	PD_-20_FEF_50_
N	484	484	484	484	484
PD-level not reached (%)	0 (0)	6 (1.2)	16 (3.3)	44 (9.1)	31 (6.4)
Diagnostic accuracy differentiating AHR from non-AHR in all patients but excluding patients not having reached PD-level					
n (%)	484 (100)	478 (98.8)	468 (96.7)	440 (90.9)	453 (93.6)
Prevalence	69.6%	69.6%	69.6%	69.6%	69.6%
Sensitivity	93.2%	89.3%	82.8%	54.9%	71.5%
Specificity	35.4%	44.2%	52.4%	85.0%	55.1%
PPV	76.8%	78.6%	79.9%	89.4%	78.5%
NPV	69.3%	64.4%	57.0%	45.1%	45.8%
Diag. Odds Ratio (DOR) [33]	7.473	6.628	5.291	6.915	3.081
95% CI of DOR	4.347–12.847	4.123–10.655	3.443–8.133	4.189–11.417	2.061–4.605
pos. LR	1.443	1.600	1.739	3.660	1.592
neg. LR	0.192	0.242	0.328	0.531	0.517
Diagnostic effectiveness	0.756	0.756	0.736	0.641	0.665
Cumulative percent-response in all patients (asthmatics and non-asthmatic subjects) at each provocation level n/n (%)					
1^st^ PD level (%)	161/484 (42.4)	126/484 (26.0)	94/484 (19.4)	94/484 (9.7)	99/484 (20.5)
2^nd^ PD level (%)	362/484 (74.8)	326/484 (67.4)	296/484 (61.2)	217/484 (44.8)	280/484 (57.9)
3^rd^ PD level (%)	415/484 (85.7)	395/484 (81.6)	376/484 (77.7)	270/484 (55.8)	344/484 (71.1)
No AHR detected	104/484 (21.5)	132/484 (27.3)	164/484 (33.9)	302/484 (62.4)	206/484 (42.6)

*PD* provocation dose, *PPV* positive predictive value, *NPV* negative predictive value, *LR* likelihood ratio, *DOR* diagnostic odds ratio [33]

### Test-duration and MCH-doses to achieve PD-levels

We found it of clinical relevance to compare the test duration and MCH-doses to achieve PD-levels by the different target lung function parameters. Table 3 shows that the test duration was significantly shorter for PD_−40_sG_eff_ compared to PD_−20_FEV_1_, or PD_−20_FEF_50_ (14:45 ± 5:54 min. versus 17:46 ± 5:16 min; 16:17 ± 5:36 min respectively). Consequently, the provocation doses to which subjects are exposed to MCH were significantly lower for PD_−40_sG_eff_ compared to PD_−20_FEV_1_, or PD_−20_FEF_50_ (0.495 ± 0.491 mg versus 0.739 ± 0.615 mg; 0.625 ± 0.588, respectively).

**Table 3:** Test-duration and provocation-doses of methacholine (mg) needed to achieve the different provocation-dose levels

Comparison of average test-duration until different PD-levels reached					
	Number	Mean	SD	Lower 95%CL	Upper 95%CL
PD_40_ sG_eff_	484	14:45	05:54	14:15	15:14
PD_20_ FEV_1_	484	17:46	05:16	17:18	18:14
PD_20_ FEF_50_	484	16:17	05.36	15:47	16:47
Test-duration: sG_eff_ < FEV_1_; sG_eff_ < FEF_50_; FEF_50_ < FEV_1_ (p<0.001)					
Provocation-doses of methacholine (mg) needed to reach the different PD levels					
PD_40_ sG_eff_					
AHR	409	.495	.491	.447	.543
No AHR	75	2.185	.059	2.17	2.197
PD_20_ FEV_1_					
AHR	176	.739	.615	.647	.830
No AHR	243	2.189	.044	2.183	2.194
PD-level not reached	65	.873	.450	.761	.985
PD_20_ FEF_50_					
AHR	307	.625	.588	.559	.691
No AHR	146	2.188	.046	2.180	2.195
PD-level not reached	31	.936	.496	.754	1.117
Comparison between the 3 test procedures assessed by summary independent sample *t*-test					
		mean diff.	SE	*t*	Significance
PD_40_ sG_eff_ versus PD_20_ FEV_1_		.244	.048	5.088	*p*<.000
PD_40_ sG_eff_ versus PD_20_ FEF_50_		.130	.040	3.223	*p*=.001
PD_20_ FEV_1_ versus PD_20_ FEF_50_		.114	.056	2.011	*p*=.045

### Influence of pulmonary hyperinflation prior to or during MCT

**Fig. 3:**
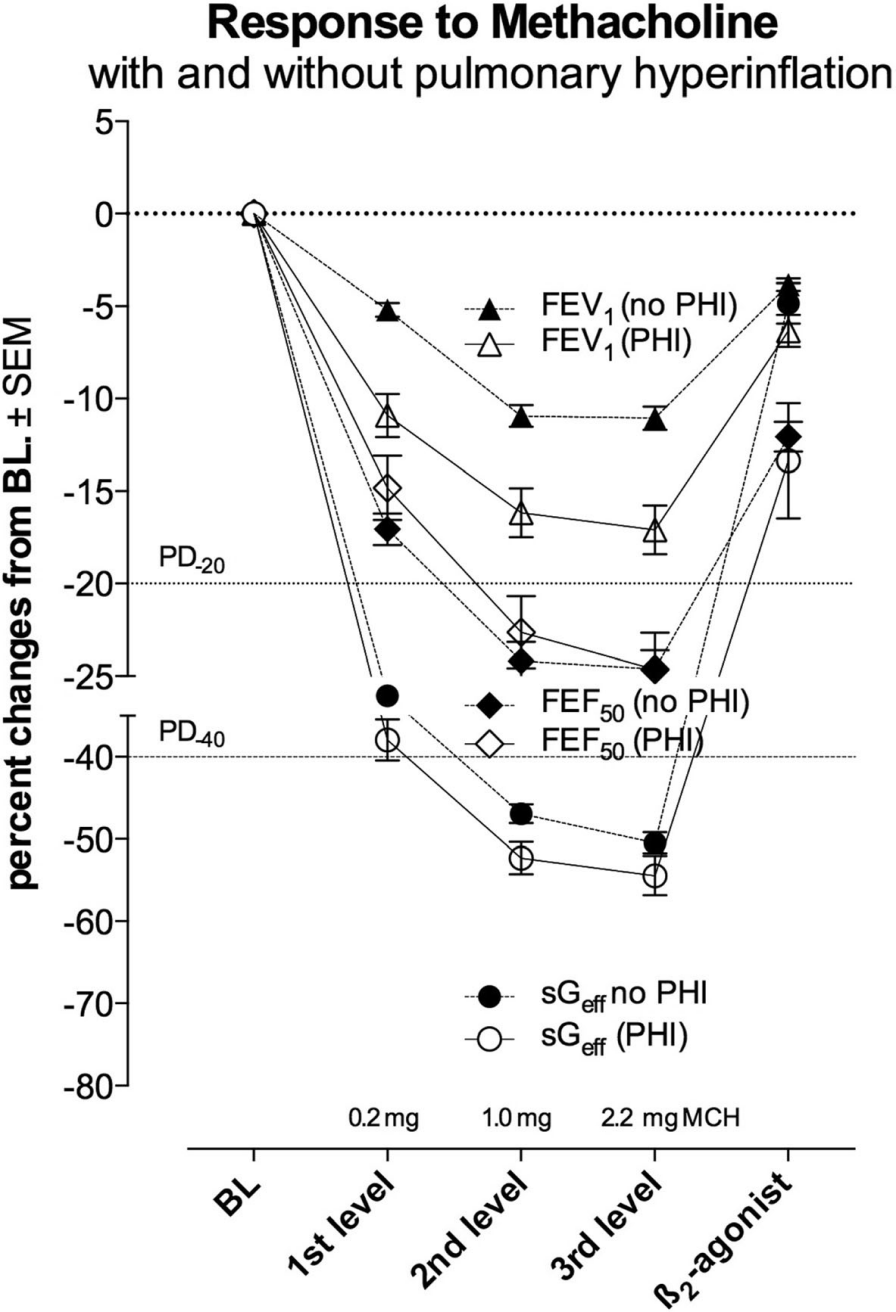
Response to methacholine in asthmatic patients comparing those with initial or developed pulmonary hyperinflation (*n* = 78;16.1%) with those without pulmonary hyperinflation (*n* = 404;83.9%), assessed by forced expiratory volume in one second (FEV_1_) and forced expiratory flow at 50% vital capacity (FEF_50_) and the effective specific conductance sG_eff_

Changes of end-expiratory lung volume (EELV) during MCT cannot be assessed by spirometry, especially not a shift of the flow-volume curve towards total lung capacity in consequence of dynamic hyperinflation, which results in an elevated residual volume and a decrease of inspiratory capacity and vital capacity as well. However, a shift of EELV toward total lung capacity may have an influence on the magnitude of FEV_1_. In 63 of 337 asthmatic patients (18.7%) pulmonary hyperinflation was observed prior to testing, or was developed during MCT. In Fig. 3 response to MCH is compared in all subjects differentiating those with pulmonary hyperinflation which those without pulmonary hyperinflation. There was a much more pronounced AHR in patients with pulmonary hyperinflation (mean diff in PD_−40_sG_eff_: 7.5%, n.s.; PD_−20_FEV_1_: 5.8% *p* < 0.001; PD_−20_FEF_50_: 7.6%, n.s.). Noteworthy to realise that pulmonary hyperinflation influenced AHR measured by PD_−20_FEV_1_ significantly. Moreover interestingly, differences were not significant for PD_−40_sG_eff_. This may be a formal proof, that the assessment of AHR using the integral method of sG_eff_, evaluates changes of airway mechanics concomitantly with changes of EELV during MCT.

### Receiver operating characteristics

**Fig. 4:**
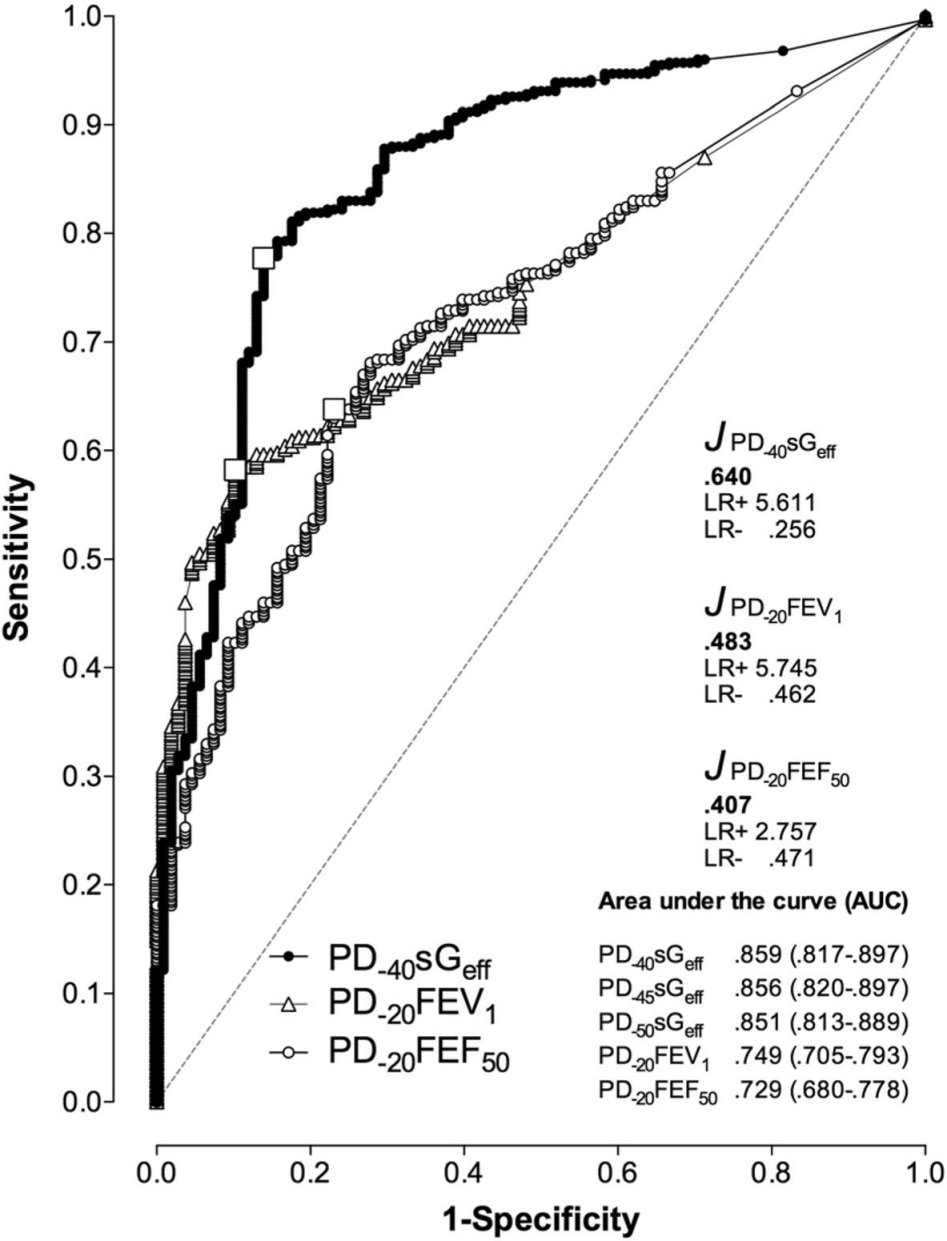
Receiver operating curves (ROC) describing the relationship between sensitivity and specificity of percent changes obtained at the corresponding provocation dose levels, PD_-40_sGeff for a 40% fall in effective specific conductance, PD_-20_FEV_1_ for a 20% fall in forced expiratory volume in one second, and PD_-20_FEF_50_ for a 20% fall in forced expiratory flow at 50% vital capacity during methacholine challenge represented by methacholine

Receiver operating curves (ROC) describing the relationship between sensitivity and specificity of percent changes during MCT of the 3 lung function parameters are given in Fig. 4. The area under the curve (AUC) for absolute change of sG_eff_ yielded the greatest value for PD_−40_sG_eff_ (.859; 95% CI: .857-.897) compared to PD_−45_sG_eff_ and PD_−50_sG_eff_ on one hand, and compared to PD_−20_FEV_1_ (.749; 95% CI: .705-.793) and PD_−20_FEF_50_ (.729;) 5% CI: .680-.778) on the other hand. Considerable differences are also found regarding the Youden J-index and the likelihood ratios, both estimates of diagnostic test performance.

## Discussion

### Key findings

The principal goal of our study was to demonstrate that the performance of MCTs using the plethysmographic technique in addition to the spirometric approach offers some fundamental benefits, such as independence from deep inspiration, and hence, modulation of the airway calibre [10, 31–36], avoidance of forced expiratory manoeuvres, and hence the subject’s cooperation and coordination, which is known to change the airway responsiveness during the test procedure [34, 37, 38]. There are two important denouements, which are achieved, if the spirometric assessment of AHR is combined with whole-body plethysmography. First, MCTs based on a plethysmographic approach offers improved diagnostic accuracy. Second, the broncho-provocating process by MCH in relation to the development of pulmonary hyperinflation and/or the phenomenon of dysanapsis, the ratio FEF_25–75_/FVC thought to be a surrogate measure of airway size relative to lung size significantly associated with AHR [44]. The present study positively confirms the findings of Nensa et al. [10] reinforcing sR_eff_, and its reciprocal value sG_eff_ respectively, as the most useful target parameters in the detection of AHR. Due to our observations of much higher percent-response to MCH by PD_−40_sG_eff_ than by PD_−20_FEV_1_, we suggest, that some false negative PD_−20_FEV_1_ tests resulted in an underestimation of the severity of AHR, and hence potentially missed the diagnosis of asthma. In the following discussion we would like to focus on some important aspects of MCT performed by whole-body plethysmography, if combined with the spirometric assessment. Furthermore, we evaluate the physiological aspects of the so-called dysanapsis [13, 14], which was found to be an important determinant of AHR to MCH, and hence airway narrowing during MCT by different author-groups [15–17, 45].

### Diagnostic accuracy

There is an increasing interest to define proximity of measurement results comparing different test procedures. Recently Porpodis et al. [46] compared the diagnostic validity of MCH with mannitol. Based on their spirometric PD_−20_FEV_1_-results they found that both challenge tests were equivalent in diagnosing asthma. In the present study, however, we first look at the accuracy of different target lung function parameters, which could be discriminative between “asthma” and “non-asthma”.

The most remarkable finding of the present study is that the sG_eff_ with a provocation level of PD_−40_presented the highest diagnostic accuracy (sensitivity, specificity likelihood ratio, diagnostic odds ratios, ROC curves) for the diagnosis of asthma. In comparison with PD_−20_FEV_1_ and PD_−20_FEF_50_ the response-pattern to MCH evaluated by PD_−40_sG_eff_ was consistently different. Identical agreement regarding severity of AHR was only found in 159 (32.9%) cases (44 severe AHR, 50 moderate AHR, 19 low AHR, 46 no AHR). In comparison to PD_−40_sG_eff_, AHR-responses of PD_−20_FEV_1_ were observed in 83 (24.9%) cases only one PD-level later, in another 73 (21.9%) cases only two PD-levels later, and in 20 (6.0%) cases even only three PD-levels later.

Furthermore, in contrast to PD_−40_sG_eff_, the level of PD_−20_FEV_1_was not reached in 65 cases (13.5%). It follows that in 67.1% of asthmatic patients, a disagreement regarding MCH-response between PD_−40_sG_eff_ and PD_−20_FEV_1_ was found. Since the diagnosis of asthma was primarily based on the results of AHR evaluated by PD_−20_FEV_1_ the question remains open whether or not some asthmatics are hidden in the collective of patients diagnosed as chronic cough disease. This question, however, can only be answered by a properly planned prospective study.

### Deep inspiration during MCT

It has well been recognised that deep inspirations play a major role in modulating airway calibre and airway responsiveness. In healthy subjects, deep inspirations reduce the level of pharmacologically induced airway obstruction by bronchodilation [45], whereas prohibition of taking deep breaths enhances the reaction to a broncho-constrictor agent [33]. Moreover, it has been recognised that inhalations to total lung capacity are broncho-protective, particularly in subjects with borderline AHR and non-asthmatics [34]. However, it has also been shown, that in asthmatics with mild AHR, deep inhalations do not produce a significantly lower response to MCH [37, 47] than tidal breathing [40].

### Site of action and interrelationship with dysanapsis

For many years it has been recognised that one factor determining the presence of AHR to broncho-provocative agents such as MCH is airway size. It varies from one individual to another, and some of this variability cannot be explained by differences in lung size between individuals [15, 16]. The term “dysanapsis” was initially proposed by Green et al. [13] to describe this disproportionate, but physiologically normal and gender-specific growth between airways and lung parenchyma. The wide variation in maximal expiratory flow rates between individuals with similar lung size was interpreted to mean that there is no consistent association between lung and airway size. Mead [14] determined the association between airway size and lung size in adult women and men. He found that healthy adult men have airways that are larger in diameter than the airways of women. Moreover, it was concluded that women and boys have airways that are smaller relative to lung size compared to men, and therefore, the apparent gender-based differences occur late in the growth period. These findings were confirmed by Shell et al. [48], demonstrating evidence for gender-specific dysanapsis as shown by computer tomography (CT) imaging. There are significant male–female differences in the luminal areas of the larger and central airways, which are not accounted for by differences in lung size.

Forced expiratory flow between 25 and 75% of vital capacity (FEF_25–75_) is considered as the most effort-independent part of the flow volume curve. This fraction is very sensitive regarding flow-limitation in peripheral airways, when chronic airflow obstruction is present [13]. Since direct measures of airway mechanics and lung size are often not available concomitantly, and FEF_25–75_ and forced vital capacity (FVC) are measured routinely during forced breathing manoeuvres, the ratio between FEF_25–75_ and FVC has been taken as a surrogate measure of dysanapsis [7, 13, 14, 17, 44]. Parker et al. [17] showed that when subjects were classified into four groups according to their FEF_25–75_/FVC ratio, subjects with lowest ratio also had the lowest PD_−20_FEV_1_, supporting the notion that subjects who are more sensitive to MCH have smaller airway calibres in relation to their lung size. They concluded that baseline FEF_25–75_/FVC ratio could be a determinant of AHR to MCH. Moreover, Mirsadraee et al. [7] showed that the diagnostic accuracy of PC_−20_FEF_25–75_/FVC and PD_−20_FEF_50_ were similar to PD_−35_sGaw and superior to PD_−20_FEV_1_.

The analysis in a subgroup of our asthmatic patients (*n* = 363) regarding dysanapsis expressing the FEF_25–75_/FVC ratio as age- and gender-specific quartiles showed significant association to the severity of AHR assessed by PD_−40_sG_eff_, PD_−20_FEV_1_, and PD_−20_FEF_50_. However, within these quartiles of dysanapsis the response-pattern of the 3 lung function parameters were completely different. The differences between severe and medium AHR can be much better presented by PD_−40_sG_eff_ (36.7%, 46.7%, resp.) than by PD_−20_FEV_1_ (8.2% 23.3%, resp.), or by PD_−20_FEF_50_ (23.2%, 34.9%, resp.), if related to the phenomenon of “airway narrowing” by FEF_25–75_/FVC ratios < 25%ile, 25 to 50%ile. These associations existed for both male and female asthmatics, indicating that as opposed to Gaw and sGaw, AHR assessed by sG_eff_ represents a much wider range of central and peripheral airways within the bronchial tree. In line with Parker et al. [17] we therefore conclude that AHR assessed by PD_−40_sG_eff_ is in strong association with dysanapsis, and may well provide a new conceptual approach, and hence better understanding of mechanisms that predispose an individual to asthma. Within the group of non-asthmatic subjects (*n* = 148) 14 (9.5%) (8 males, 6 females) presented with a PD_−40_sG_eff_ to be labelled as “severe”, within the subgroup of dysanapsis with a ratio of FEF_25–75_/FVC < 25%ile, and the question remains open whether in these patients the diagnosis “asthma” was missed, because the diagnosis “asthma” was only expressed when PD_−20_FEV_1_ demonstrated AHR.

### Limitations of method

The present study has some limitations. Firstly, because this was a retrospective study, there is a potential risk that some asthmatic patients might have been under-diagnosed. As standard for the diagnosis “asthma” PD_−20_FEV_1_was taken and it could well be that a certain number of patients with cough-related diseases should be attributed to the groups of “asthmatics”, if in fact PD_−40_sG_eff_indicated a moderate or high AHR, and the subject presented with a low FEF_25–75_/FVC ratio. Secondly, the database from which the evaluation was performed offered only a small number of really healthy subjects. It is not totally excluded that some “potential false positive” responses in the non-asthmatic group might have been more prevalent compared to healthy controls, and the differences between the asthmatics and false positive responders might be smaller than with healthy subjects. Thirdly, usually a retrospective study looks backwards and examines exposures to suspected risks or protection factors in relation to an outcome that is established at the start of the study. In our case–control study, however, we evaluated individual lung functions within 2 diagnostic groups, and for each parameter the chance to obtain a comprehensive result regarding AHR was the same. Great care was taken to avoid biases for one or the other parameter and confounding factors such as the development of pulmonary hyperinflation or dysanapsis were carefully addressed.

## Conclusion

Important findings in regard to the performance of MCT as a hallmark in the diagnosis of bronchial asthma could be discovered, if the spirometric approach is combined with the assessment by whole-body plethysmography. Where diagnostic accuracy is required, there are striking differences regarding the choice of target parameters. Although the spirometric assessment based on changes in FEV_1_ is regarded as the gold standard for evaluation of MCT, this study shows some important physiological inadequacies, to be considered as prerequisites, which could well be important for diagnosing “asthma”. Deep inspiration in the set-up of test-sequences should be carefully monitored, as it is inevitably linked to forced breathing manoeuvres. Using the APS technology within the Jaeger MasterLab plethysmograph, MCTs are by default based on plethysmographic tidal breathing analysis, applying breathing loops with an implementation of real-time multi-level integral computation of all data points throughout the whole breathing cycle. In this light the study demonstrates that the plethysmographic sG_eff_ features a highly sensitive and reproducible target parameter for the assessment of AHR by MCTs. Furthermore, the combined assessment of AHR by spirometry and whole-body plethysmography offers the possibility to include changes of EELV at FRC_pleth_ (and hence pulmonary hyperinflation), as well as the phenomenon of dysanapsis to be included into the differentiation between “asthmatic” and “non-asthmatics”.

## Additional files

Additional file 1: Paradigm shift in the assessment of airway mechanics. (DOCX 880 kb)
Additional file 2: The Aerosol Provocation System (APS). (DOCX 545 kb)
Additional file 3: Assessment of airway hyperreactivity (AHR) in practical terms. (DOCX 259 kb)
Additional file 4: Statistical approach. (DOCX 128 kb)

## Abbreviations

AHR: Airway hyperreactivity
APS: Aerosol provocation system
ATS: American thoracic society
AUC: Area und the curve
BL: Baseline
CD: Cumulative dose
DOR: Diagnostic odds ratio
EELV: End-expiratory lung volume
ERS: European respiratory society
FEF_25–75_: Forced expiratory flow between 25 and 75% vital capacity
FEF_50_: Forced expiratory flow at 50% of vital capacity
FEV_1_: Forced expiratory volume in 1 s
FRC_pleth_: Plethysmographic functional residual capacity
FVC: Forced vital capacity
J: Youden’s index
LR-: Negative likelihood ratio
LR+: Positive likelihood ratio
MCH: Methacholine
MCT: Methacholine challenge test
NPV: Negative predicting value
P_amb_: Barometric pressure
PD: Provocation doses
PD_−20_FEF_25–75_: Provocation dose at which the forced expiratory flow between 25 to 75% of forced vital capacity decreases at least 20% from baseline
PD_−20_FEV_1_: Provocation dose at which the forced expiratory volume in 1 s decreases at least 20% from baseline
PD_−40_sG_eff_: Provocation dose at at which the effective specific airway conductance decreases at least 40% from baseline
PD_−45_sG_eff_: Provocation dose at which the effective specific airway conductance decreases at least 45% from baseline
PD_−50_sG_eff_: Provocation dose at which the effective specific airway conductance decreases at least 50% from baseline
P_H2O_: Saturated vapour water pressure
PPV: Positive predicting value
R_aw_: Airway resistance (two-angle technique)
ROC: Receiver operating curve
sG_aw_: Specific airway conductance (two-angle technique)
sG_eff_: Effective specific airway conductance (integral technique)
sR_aw_: Specific airway resistance (two-angle technique)
sR_eff_: Effective specific airway resistance (integral technique)
sWOB: Effective resistive work of breathing
V’: Flow
∆ V_box_: Volume displacement of the plethysmographic box
V_T_: Tidal volume
