# Supplementary material for: Diagnostic accuracy of methacholine challenge tests assessing airway hyperreactivity in asthmatic patients - a multifunctional approach
Source: Respir Res. 2016 Nov 17;17:154. doi: 10.1186/s12931-016-0470-0 (PMC5114725; doi:10.1186/s12931-016-0470-0)
Supplement: Additional file 1: — Paradigm shift in the assessment of airway mechanics. (DOCX 880 kb) [file 12931_2016_470_MOESM1_ESM.docx]

Additional File 1: Paradigm shift in the assessment of airway mechanics

Two-point angle-approach [1, 2]

Until recently, airway resistance (Raw) was calculated using the two-point angle-approach, unavoidably linked to an average amount of FRC_pleth_ given by the equation:

$${Raw=\frac{sRaw}{{FRC}_{pleth}+\frac{V_{T}}{2}}=\frac{{\Delta V}_{box}}{V'}\cdot\frac{P_{amb}- P_{H2O} sat}{{FRC}_{pleth}+\frac{V_{T}}{2}}}$$

where *V_T_* is the tidal volume, *∆V_box_* is the change of the displacement-volume within the plethysmograph, also named “shift volume”, i.e. the volume which is lost in compression and originated in decompression during the breathing cycle, *V’* the flow in the mouth piece, *P_bar_* the barometric pressure, *P_H2O_* the partial pressure of water vapour saturated in the lungs. It must be recognised, that the higher FRC_pleth_ is (in the presence of pulmonary hyperinflation), the lower Raw is calculated using this fixed parameter as the denominator.

By algebraic manipulation of the formula, Dab et al. [3, 4] proposed a different approach, which precludes the measurement of FRC_pleth_, thereby facilitating measurements of sRaw, especially in young children. The equations have been simplified as follows:

$$sRaw= \frac{\Delta V_{box}}{V'}*(P_{amb}- P_{H2O})$$

It follows, that in such an approach sRaw is measured regardless of the EELV, which bias the value of sRaw, if pulmonary hyperinflation or lung restriction of the patient is present.

Integrative assessment of airway behaviour by extended dimensional analysis throughout the entire tidal breathing circle [5-7]

Although numerous parameters of airway resistances can be calculated from plethysmographic measurements [8], the most promising approach was proposed by Matthys and Orth [6], defining the so called “specific effective resistance” (sR_eff_) computed as the ratio of the integral of the area of the plethysmographic *box-volume shift versus tidal-volume* as numerator, and the integral of the area of the *tidal flow versus volume (flow-volume loop)* as denominator throughout the entire respiratory cycle. The appropriate equation is as follows:

$${sR}_{eff}= \frac{\left( P_{amb}- P_{H2O} \right)* \oint{\Delta V}_{box} dV}{\oint V'dV}$$

where P_amb_ is the barometric pressure, the integral $\oint\Delta V_{box}dV$ an equivalent to the area enclosed by the resistive work of tidal breathing (sWOB) and the integral $\oint V©dV$ an equivalent to the area of the tidal flow-volume loop [9]. Matthys et al. [5, 6] extend the calculation to the dimensional analysis applied by Jaeger and Otis [7], in order to integrate effects of variable flows and nonlinearities of mouth flow-shift volume loops during tidal breathing. By such an approach an averaged airway resistance can be obtained, which is independent from the EELV at FRC_pleth_. The outstanding characteristics of such measurements of airway mechanics, is its reflection of an integrative assessment of airway behaviour throughout the entire tidal breathing circle. Real-time acquisition and digital integration of the respective resistance loop is representative for the whole breathing cycle incorporating all (not just two) sample points of the entire airway resistance loop, and improves the signal-to-noise ratio. Since flows are lower and more variable in infants and children, the assessment of sR_eff_ is thought to be more accurate than total airway resistance or resistance taken from peak pressure points in such subjects [9]. What was previously anticipated for the measurements of specific airway resistance (sRaw) regarding strength, repeatability and inter-dependence from the breathing pattern [10-12], is much better achieved by sR_eff_ [13].

Regarding the topical representation of airway patency it is anticipated that the parameters of airway mechanics assessed by the two-point technique (sRaw and sGaw), and the parameters obtained by the integral method (sR_eff_ and sG_eff_) assess airway mechanics firstly down to the 9^th^ to 12^th^ airway generations. Secondly, however, sR_eff_ and sG_eff_ assess airway mechanics also in relation to the lung volume (FRC_pleth_), and hence are representative for the small and peripheral airways of generations down to the 24^th^ generation. It follows that sR_eff_ and sG_eff_ do incorporate central and peripheral airways, e.g. represent the entire bronchial tree [1]. There is no other lung function parameter, which allows the same complete assessment of the lung. The assessment of sR_eff_ and sG_eff_ is comparable to the FEV_1_, the latter, however, largely dependent from the subject’s cooperation, whereas the former parameters can be measured during tidal breathing, which is an advantage especially in children, elderly persons and patients with advanced lung involvement.

Synoptic demonstration between the specific resistive work of breathing (sWOB) and the tidal flow-volume loop as product of airway mechanics

Assessment of airway mechanics, especially sR_eff_, its reciprocal value the sG_eff_, are focused on to integrals computed throughout the whole respiratory cycle of concomitant changes in the volume-pressure and flow-pressure relationship. By this integration the resistive tidal work of breathing (sWOB) is set in relation to the product given by the tidal flow-volume loop. Since this technique was already incorporated in the MasterLab infant plethysmograph (version 4.0), details can comprehensibly been explained best by a **Print-Screen ,**originally depicted from the infant whole-body plethysmograph (Figure)[14], demonstrating breath by breath tracings, from which sR_eff_, its reciprocal values sG_eff_ resp. are computed.


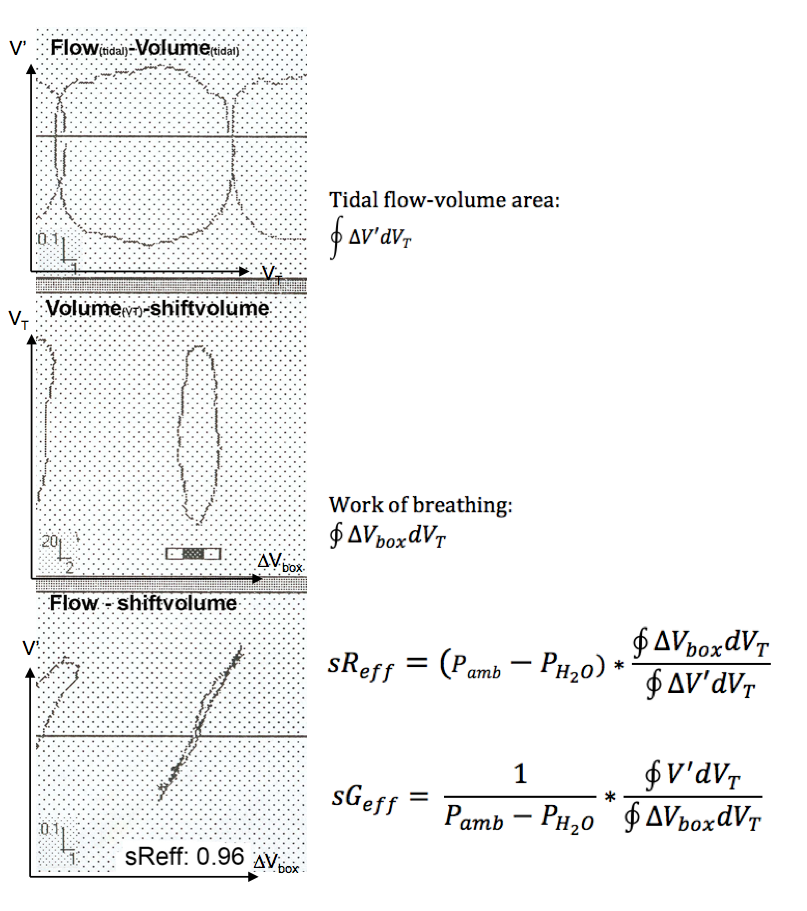


**Figure:** Breath tracing obtained by the MasterLab infant whole-body plethysmograph
 (Vers. 4) [14]

Physiologic background
http://ww.atsjournals.org/ doi/suppl/ IO.1164 / ree m. 200407-94 SOC/ suppl_file/ on line_methods.pdf

The total work of breathing is the integral of the pressure in the lung (p_L_) over volume for the whole breathing cycle:

$$Wb=\oint p_{L}dV$$

where p_L_ is determined by the volume-change of the box signal and the volume of the lung (FRC_pleth_).

∆V is the volume change in the body box, P_amb_ the ambient pressure and FRC_pleth_ the volume of end-expiratory resting level. The volume changes are measured relative to an arbitrary start volume, start pressure (P_0_), where p_L_ is the sum of the p_0_, the elastic recoil component (E), defined as ∆p_L_ / ∆V and the resistive pressure R.V’. It follows:

$$\begin{matrix} \oint pdv=\oint p_{0}dv+E.\oint VdV+R.\oint dV \\ \oint p_{0}dV=p_{0}.(V_{end}-V_{start})=zero \end{matrix}$$

when integrated over a whole breathing cycle and assuming that lung volume remains constant between beginning and end of the breathing cycle. Moreover,

when integrated over a whole breathing cycle and assuming that lung volume remains constant between beginning and end of the breathing cycle.

Therefore, the resistive component can be left and we obtain

This yields the definition of the specific effective resistance:

The integral is equivalent to the area enclosed by the specific work of breathing loop and the integral $\oint V©dV$ is equivalent to the area of the flow -volume-loop. Both loops are measured during tidal breathing. The ratio of these two integrals, which are obtained by real–time data acquisition, is equivalent to the calculation of the slope through all (not just two) sample points. Additionally, inspiratory and expiratory sR_eff_ can be calculated from the loops split by the zero-flow line, the latter given by the zero-flow point of the work of breathing loop. Since flows are lower and more variable in children, the assessment of sR_eff_ is more accurate than total airway resistance or resistance taken from peak pressure points and, hence may be preferentially used in children and even in infants [9].

References

1. Goldman M, Smith HJ, Ulmer WT. Lung function testing: Whole-body plethysmography. European Respiratory Society Monograph 2005; 31: 15-43.

2. Criee CP, Sorichter S, Smith HJ, Kardos P, Merget R, Heise D, Berdel D, Kohler D, Magnussen H, Marek W, Mitfessel H, Rasche K, Rolke M, Worth H, Jorres RA, Working Group for Body Plethysmography of the German Society for P, Respiratory C. Body plethysmography--its principles and clinical use. Respir Med 2011; 105: 959-971.

3. Dab I, Alexander F. A simplified approach to the measurement of specific airway resistance. Pediatr Res 1976; 10: 998-999.

4. Dab I, Alexander F. On the advantages of specific airway resistance. Pediatr Res 1978; 12: 878-881.

5. Matthys H, Keller R, Herzog H. Plethysmographic assessment of trapped air in man. Respiration 1970; 27: 447-461.

6. Matthys H, Orth U. Comparative measurements of airway resistance. Respiration 1975; 32: 121-134.

7. Jaeger MJ, Otis AB. Measurement of Airway Resistance with a Volume Displacement Body Plethysmograph. J Appl Physiol 1964; 19: 813-820.

8. Springer C, Vilozni D, Bar-Yishay E, Avital A, Noviski N, Godfrey S. Comparison of airway resistance and total respiratory system resistance in infants. Am Rev Respir Dis 1993; 148: 1008-1012.

9. Jackson AC, Tennhoff W, Kraemer R, Frey U. Airway and tissue resistance in wheezy infants: effects of albuterol. Am J Respir Crit Care Med 1999; 160: 557-563.

10. Aurora P, Bush A, Gustafsson P, Oliver C, Wallis C, Price J, Stroobant J, Carr S, Stocks J. Multiple-breath washout as a marker of lung disease in preschool children with cystic fibrosis. Am J Respir Crit Care Med 2005; 171: 249-256.

11. Lowe L, Murray CS, Custovic A, Simpson BM, Kissen PM, Woodcock A. Specific airway resistance in 3-year-old children: a prospective cohort study. Lancet 2002; 359: 1904-1908.

12. Subbarao P, Hulskamp G, Stocks J. Limitations of electronic compensation for measuring plethysmographic airway resistance in infants. Pediatr Pulmonol 2005; 40: 45-52.

13. Kraemer R, Blum A, Schibler A, Ammann RA, Gallati S. Ventilation inhomogeneities in relation to standard lung function in patients with cystic fibrosis. Am J Respir Crit Care Med 2005; 171: 371-378.

14. Kraemer R. Whole-body plethysmography in the clinical assessment of infants with bronchopulmonary diseases. Respiration 1993; 60: 1-8.
