# Supplementary material for: Diagnostic accuracy of methacholine challenge tests assessing airway hyperreactivity in asthmatic patients - a multifunctional approach
Source: Respir Res. 2016 Nov 17;17:154. doi: 10.1186/s12931-016-0470-0 (PMC5114725; doi:10.1186/s12931-016-0470-0)
Supplement: Additional file 2: — The Aerosol Provocation System (APS). (DOCX 545 kb) [file 12931_2016_470_MOESM2_ESM.docx]

Additional file 2: The Aerosol Provocation System (APS)
(CareFusion, Würzburg, Germany)

Apart from the technical improvements of the plethysmographic technique the integration of the Aerosol Provocation System (APS) in the Jaeger MasterScreen plethysmograph (CareFusion, Würzburg, Germany) previously described [1-3], features additional advantages in so far as the production of aerosol using a jet-type nebuliser (Sidestream, Philips/Respironics) powered by pressure-controlled compressed air is computer-controlled, the dosimeter control being an essential part of the lung function measurement software. Moreover, the APS is linked with a filter-system, absorbing the methacholine in the exhaled air, which reduces contamination of environment and undesirable exposure to the technician. Moreover, the initial integrated pressure calibration procedure of the compressor ensures highly constant and reproducible nebuliser output. APS was calibrated to produce a continuous nebuliser output of 240 mg/min with a mean particle size of 3.2 μm. The duration of aerosol bolus delivery was chosen in a narrow range from 0.3 to 0.6 s. Subjects placed their lips around the mouthpiece during the whole time at each challenge level in sitting position during plethysmographic and spirometric measurements. When inhalation became steady, the APS side-stream is triggered 0.1 s after the start of inspiration measuring drug inhalation during quiet breathing controlled by the APS.


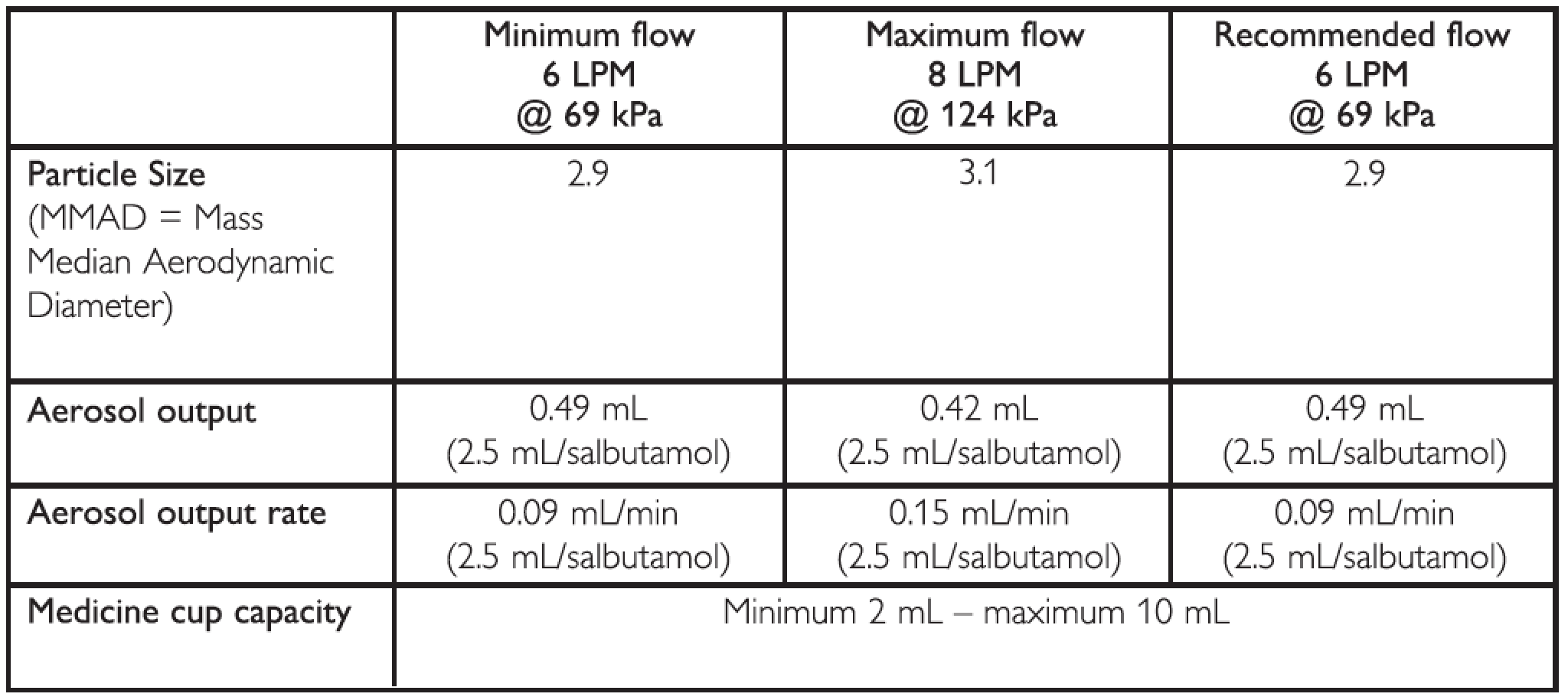

**Table:** Technical characteristics in relation to 2 different flows of the Sidestream nebulizer (Philips Respironics)

The APS was initially evaluated regarding particle size, aerosol output, and aerosol output rate (Table) as well as cumulative Drug distribution of salbutamol (Figure) by the manufacturer.


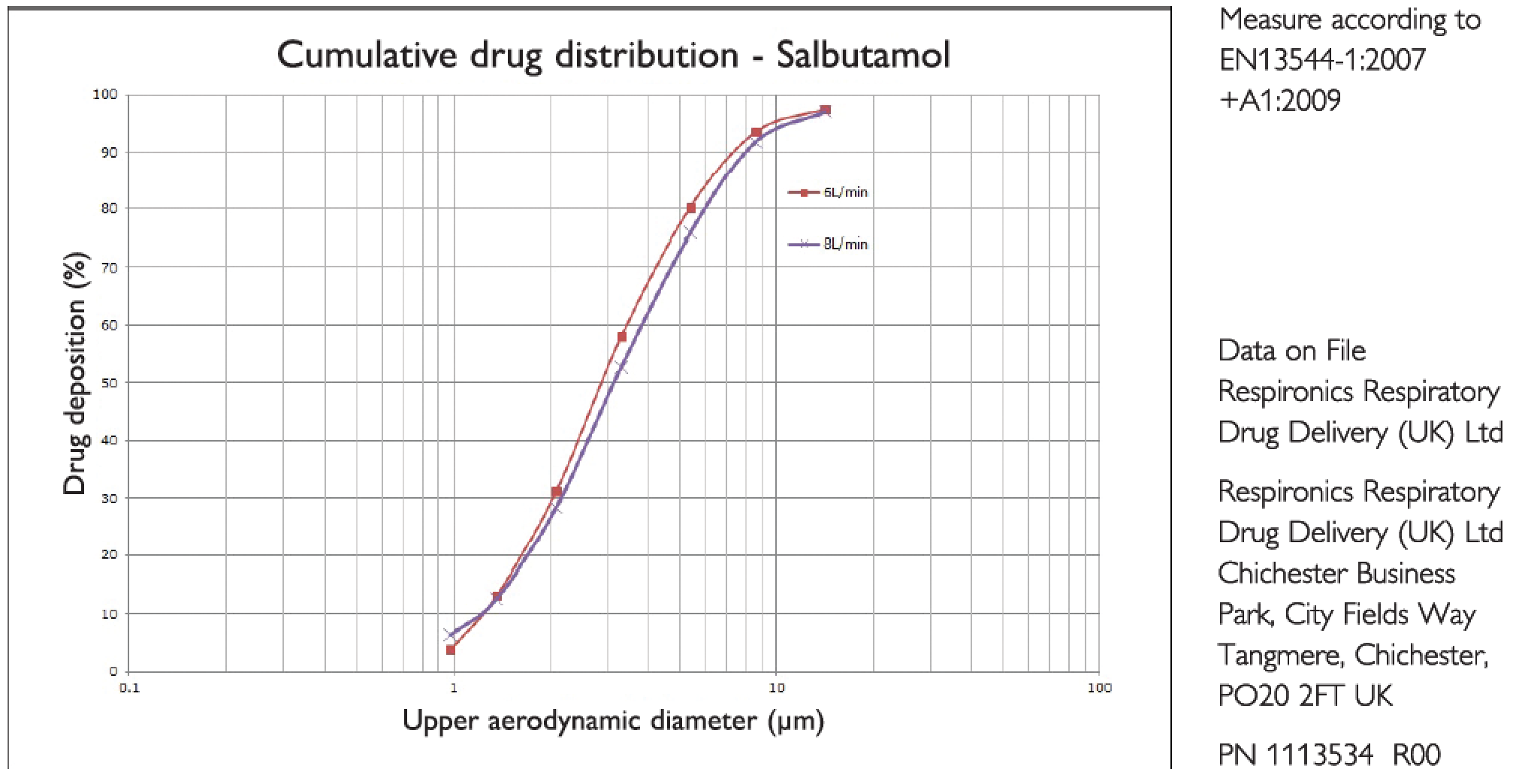


**Figure**

Drug deposition (%) in relation to aerodynamic diameter (µm) for Salbutamol

Most recently, Kannan et al. [4] performed a study looking at the fidelity of intra-bronchial deposition of aerosols by computational simulations over several breathing cycles, applying different particle sizes. This paper deals with computational methods to develop and implement accurately a quantification of the transport, deposition, and clearance of particles (range of interest: 2 to 10 μm) in the human respiratory tract. In relation to our technical approach with the APS, the authors concluded, that in order to circumvent the effect of Brownian motion the particle size should be greater than 2 µm, a condition, which is entirely fulfilled by our system.

References

1. Schulze J, Rosewich M, Riemer C, Dressler M, Rose MA, Zielen S. Methacholine challenge--comparison of an ATS protocol to a new rapid single concentration technique. Respir Med 2009; 103: 1898-1903.

2. Merget R, Jorres RA, Heinze E, Haufs MG, Taeger D, Bruning T. Development of a 1-concentration-4-step dosimeter protocol for methacholine testing. Respir Med 2009; 103: 607-613.

3. Schulze J, Smith HJ, Fuchs J, Herrmann E, Dressler M, Rose MA, Zielen S. Methacholine challenge in young children as evaluated by spirometry and impulse oscillometry. Respir Med 2012; 106: 627-634.

4. Kannan R, Guo P, Przekwas A. Particle transport in the human respiratory tract: formulation of a nodal inverse distance weighted Eulerian-Lagrangian transport and implementation of the Wind-Kessel algorithm for an oral delivery. Int J Numer Method Biomed Eng 2016; 32.
