# Supplementary material for: Diagnostic accuracy of methacholine challenge tests assessing airway hyperreactivity in asthmatic patients - a multifunctional approach
Source: Respir Res. 2016 Nov 17;17:154. doi: 10.1186/s12931-016-0470-0 (PMC5114725; doi:10.1186/s12931-016-0470-0)
Supplement: Additional file 3: — Assessment of airway hyperreactivity (AHR) in practical terms. (DOCX 259 kb) [file 12931_2016_470_MOESM3_ESM.docx]

Additional file 3: Assessment of airway hyperreactivity (AHR) in practical terms

In the Figure, a typical printout of the time-response curves for FEV_1_, MEF_50_ and sG_eff_ obtained in a female asthmatic patient (E.J. age 37 years) are presented, showing that airway reactivity is much quicker and stronger represented by sG_eff_, than FEV_1_ and/or MEF_50_. For PD_-50_sG_eff_ the cumulative Dose (CD) on level P_1_ (at time 14:26 min) was computed as 0.29 mg MCH (moderate AHR). PD_-20_FEV_1_, however gave a CD on level P_3_ (at time 21:46 min) calculated as 1.04 mg MCH (mild AHR), and PD_-20_MEF_50_ on level P_2_ (at time 18:05 min) as 0.36 mg MCH (moderate AHR). By all three parameters the clinical diagnosis “bronchial asthma” could be confirmed. However, depending by which lung function parameter, AHR could be attributed completely

**
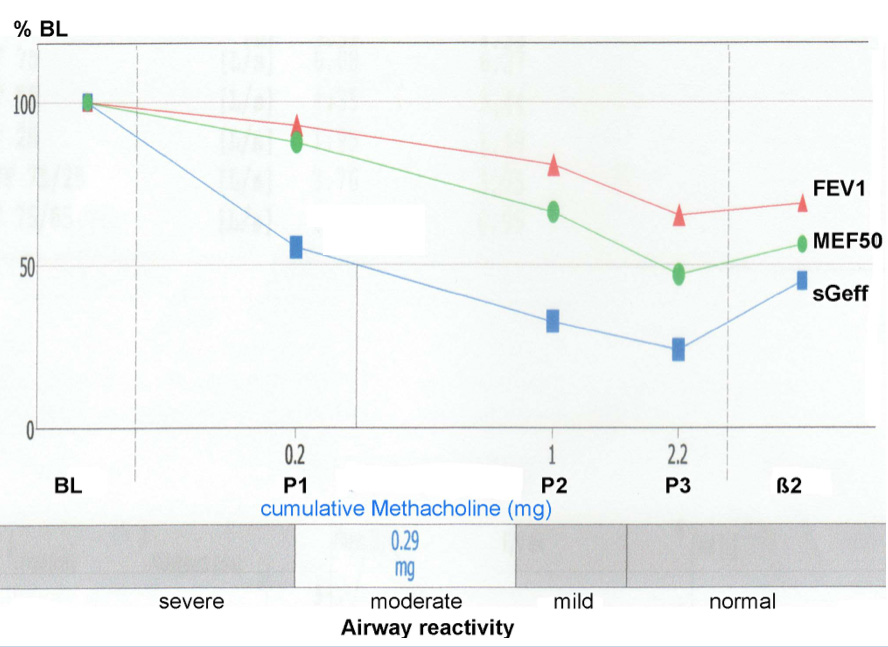
**

**Figure.** Printout of time-response curves for FEV_1_, MEF_50_ and sG_eff_ obtained in a

female asthmatic patient (E.J. age 37 years). P1, P2, P3: 1^st^, 2^nd^ and 3^rd^

provocation levels for 0.2 mg, 1.0 mg and 2.2 mg Methacholine; ß_2_:

reversibility test with 2 puffs of salbutamol

differently. Major advantages of this system are the real-time visualisation of dose administration and breathing pattern. The system measures the effective nebulisation time and calculates the cumulative dose of the required inspiratory breaths, and hence

determines in real-time exactly the cumulative administered dose MCH, referring it to the drug concentration and nebuliser power. Subjects were instructed to inhale by normal breathing from the nebuliser with a flow below 0.5 L/s. In continuous nebulisation mode, the inhalation time and number of breaths were dependent upon the concentration of the employed MCH and the breathing pattern of the subject.
