# Supplementary material for: Diagnostic accuracy of methacholine challenge tests assessing airway hyperreactivity in asthmatic patients - a multifunctional approach
Source: Respir Res. 2016 Nov 17;17:154. doi: 10.1186/s12931-016-0470-0 (PMC5114725; doi:10.1186/s12931-016-0470-0)
Supplement: Additional file 4: — Statistical approach. (DOCX 128 kb) [file 12931_2016_470_MOESM4_ESM.docx]

Additional file 4: Statistical approach

(Table 2)

The discriminative power of each lung function parameter was evaluated by measures of diagnostic accuracy such as sensitivity (Se), specificity (Sp), positive predictive values (PPV), negative predictive values (NPV), likelihood ratios, the area under receiver operating curves (ROC), the Youden's index (J) and diagnostic odds ratio (DOR). By these different diagnostic procedures different aspects of diagnostic accuracy, such as predictive ability and/or discriminative property of the MCT could be evaluated [1].

Sensitivity (Se) and specificity (Sp)
The ability to completely discriminate between subjects with different degrees of AHR, and therefore to test whether or not the cut-offs defining the provocation dose (PD) levels are really differentiating between asthmatic patients and non-asthmatic subjects was achieved by computation of Se and Sp. Significantly, since values below the cut-off of PD levels are not always indicative of AHR, false positive values (FP) have to be considered. Alternatively, those subjects presenting with values above the cut-off of PD levels, but clinically established as asthmatics, have to be regarded as false negative values (FN). Sensitivity was then expressed in percentage, defineing the proportion of true positive subjects with AHR in a total group of subjects with asthma (TP/TP+FN). Sensitivity actually expresses the potential of a test to recognise subjects with a disease. In our approach specificity, as a measure of the diagnostic accuracy , in conjunction with sensitivity, defines the proportion of non-asthmatic subjects with negative test results (no AHR) within the total number of subjects without asthma (TN/TN+FP), relating to the aspect of diagnostic accuracy that describes the tests ability to recognise subjects without asthma. However, Se, and Sp are influenced by the disease prevalence, meaning that results from one study could easily be transferred to some other setting with a different prevalence of the disease in the population.

Predictive values
Positive predictive value (PPV) represents the proportion of asthmatics with positive test result (AHR) in the total of subjects with positive result (TP/TP+FP). Negative predictive value (NPV) defines the proportion of non-asthmatic subjects with a negative test result (no AHR) in the total of subjects with negative test results (TN/TN+FN). Prevalence affects PPV and NPV differently. PPV is increased, while NPV decreased with the increase of the prevalence of the disease in a population. Whereas the change in PPV is more substantial, NPV is influenced somewhat less by the disease prevalence.

Likelihood ratio (LR**)**In the present study the likelihood ratio was defined as the ratio of a positive MCT in subjects with asthma to the non-asthmatic subjects. As such, LR directly links the pre-test and post-test probability of asthma in a specific patient [2]. Simplified, the LR indicates how many times more likely a particular MCH challenge test result will be found in an asthma population than in one without asthma. The likelihood ratio for positive test results (LR+) indicates how much more likely the positive test result is to occur in subjects with asthma compared to those without asthma. LR+ is calculated according to the following formula: LR+ = sensitivity / (1-specificity). Thus LR+ is the best indicator for ruling-in the diagnosis of asthma. The higher the LR+ the more indicative is the MCH challenge test in diagnosing asthma. A significant contribution to the diagnosis is concluded if the LR+ is higher than 10. The likelihood ratio for a negative test result (LR-) represents the ratio of the probability that a negative result will occur in subjects with asthma to the probability that the same result will occur in non-asthmatic subjects. Therefore, LR- indicates how much less likely the negative test result is to occur in an asthmatic patient than in a non-asthmatic subject. LR- is calculated according to the following formula: LR- = (1-sensitivity) / specificity. LR- is a good indicator for ruling-out the diagnosis of asthma. Good diagnostic tests have LR-< 0,1. The lower the LR- the more significant the contribution of the MCH challenge test is in ruling-out, i.e. in lowering the posterior probability of the subject having asthma. Since both specificity and sensitivity are used to calculate the likelihood ratio, it is clear that neither LR+ nor LR- are independent of the disease prevalence in examined groups. Consequently, the likelihood ratios from one study could be applicable to some other clinical setting, as long as the definition of the disease is not changed. If the way of defining the disease varies, none of the calculated measures will apply in some other clinical context.

Diagnostic effectiveness
Another global measure of diagnostic accuracy is so called diagnostic effectiveness, expressed as a proportion of correctly classified subjects (TP+TN) among all subjects (TP+TN+FP+FN). diagnostic effectiveness is also affected by the disease prevalence. With the same sensitivity and specificity, diagnostic effectiveness of a particular test increases as the disease prevalence decreases. However, this does not mean that the test is better, if in a population with low disease prevalence this test is applied. It only means that in absolute number the test gives more correctly classified subjects.

Diagnostic odds ratio (DOR)
Diagnostic odds ratio is a global measure of diagnostic accuracy, used for general estimation of the discriminative power of diagnostic procedures and could be used for the comparison of diagnostic accuracies between two or more lung function parameters used as diagnostic tests. The rationale for DOR is that it is a single indicator of test performance (like accuracy and Youden's J statistic), but which is **independent of prevalence** (unlike accuracy. Generally, DOR of a test is the ratio of the odds of positivity in subjects with disease relative to the odds in subjects without disease [3]. It is calculated according to the formula: DOR = (TP/FN)/(FP/TN). DOR depends significantly on the sensitivity and specificity of a test. A test with high specificity and sensitivity with a low rate of false positives and false negatives has a high DOR. With the same sensitivity of the test, DOR increases with the increase of the test specificity. DOR does not depend on disease prevalence; however like sensitivity and specificity it depends on criteria used to define disease and its spectrum of pathological conditions of the examined group (disease severity, phase, stage, comorbidity etc.). The diagnostic odds ratio ranges from zero to infinity, although for useful tests it is greater than one, and higher diagnostic odds ratios are indicative of better test performance [3]. Diagnostic odds ratios less than one indicate that the test can be improved by simply inverting the outcome of the test – the test is in the wrong direction, while a diagnostic odds ratio of exactly one means that the test is equally likely to predict a positive outcome whatever the true condition – the test gives no information.

ROC curve

(Figure 3)

There was a pair of diagnostic sensitivity and specificity values for every individual cut-off, and if the 1-specificity was plotted against sensitivity the shape of a ROC curve and the area under the curve (AUC) are estimates of how high the discriminative power of the lung function parameter, evaluating the MCH challenge was. The closer the curve was located to the upper-left hand corner and the larger the AUC was, the better the test parameter was discriminating between asthma and non-asthmatic subjects. The AUC can have any value between 0 and 1 and it is generally in most settings a good indicator of the merit of the particular test parameter. An AUC between 0.6 and 0.7 is considered as rather poor than sufficient, between 0.7-0.8 as fair, and between 0.8 and 0.9 as good. By the comparison of the areas under two ROC curves it could be estimated which one of two tests is more suitable for distinguishing asthma patients from non-asthmatic subjects. Most importantly, this comparison should not be based on visual or intuitive evaluation [4]. For this reason statistical analysis, which evaluates the statistical significance of the estimated difference between two AUC, is mandatory.

Youden's index (J**)**

(Figure 3)

Youden's index is one of the oldest measurements of diagnostic accuracy [5]. It is also a global measure of a test’s performance, used for the evaluation of the overall discriminative power of a diagnostic procedure and for comparison of this test with other tests. The Youden's index is calculated by deducting 1 from the sum of a test’s sensitivity and specificity expressed not as percentage but as a part of a whole number: (sensitivity + specificity) – 1. For a test with poor diagnostic accuracy, Youden's index equals 0, and in a perfect test Youden's index equals 1. Youden's index is not sensitive to differences in the sensitivity and specificity of the test, which is its main disadvantage. Namely, a test with sensitivity 0,9 and specificity 0,4 has the same Youden's index (0,3) as a test with sensitivity 0,6 and specificity 0,7. Therefore, the Youden’s index is not indicative of comparable diagnostic accuracy. If one is to assess the discriminative power of a test solely based on Youden's index, it could be mistakenly concluded that these two tests are equally effective. Youden’s index is not affected by the disease prevalence, but it is affected by the spectrum of the disease, as are also sensitivity specificity, likelihood.

Approach to compare Diagnostic Test Accuracy

(Table 3)

Comparisons between response rates obtained by the different lung function parameters in each subject, with estimates of 95% confidence intervals (CIs), were made using the χ2 test. Moreover, comparison of paired binary data obtained by the different lung function parameters were evaluated using the McNemar test without Yates correction, and using a SPSS macro routine given by <http://www.how2stats.net>. This macro was initially written by Marta Garcia-Granero, and the confidence intervals are based on a procedure described by Newcombe, R.C.[6].

Comparison of MCH-doses needed to reach PD levels for each lung function parameter (Table 3)

For that purpose the independent samples t-test was applied in order to compare the means of methacholine doses needed to perform the challenge evaluated by two independent parameter each (PD_40_ sG_eff_ versus PD_20_ FEV_1_, PD_40_ sG_eff_ versus PD_20_MEF_50_, and PD_20_FEV_1_ versus MEF_50_ as dichotomous variables, in order to determine whether there is statistical evidence that the associated parameter means needed significantly different doses of methacholine.

References

1. Irwig L, Bossuyt P, Glasziou P, Gatsonis C, Lijmer J. Designing studies to ensure that estimates of test accuracy are transferable. BMJ 2002; 324: 669-671.

2. Deeks JJ, Altman DG. Diagnostic tests 4: likelihood ratios. BMJ 2004; 329: 168-169.

3. Glas AS, Lijmer JG, Prins MH, Bonsel GJ, Bossuyt PM. The diagnostic odds ratio: a single indicator of test performance. J Clin Epidemiol 2003; 56: 1129-1135.

4. Obuchowski NA, Lieber ML, Wians FH, Jr. ROC curves in clinical chemistry: uses, misuses, and possible solutions. Clin Chem 2004; 50: 1118-1125.

5. Youden WJ. Index for rating diagnostic tests. Cancer 1950; 3: 32-35.

6. Newcombe RG. Interval estimation for the difference between independent proportions: comparison of eleven methods. Stat Med 1998; 17: 873-890.
